# Single Plasmonic Structure Enhanced Dual-band Room Temperature Infrared Photodetection

**DOI:** 10.1038/s41598-018-20028-6

**Published:** 2018-01-24

**Authors:** Jinchao Tong, Landobasa Y. M. Tobing, Yu Luo, Dawei Zhang, Dao Hua Zhang

**Affiliations:** 1 School of Electrical and Electronic Engineering, Nanyang Technological University, 50 Nanyang Avenue, 639798 Singapore; 2 Ministry of Education and Shanghai Key Lab of Modern Optical System, University of Shanghai for Science and Technology, 516 Jungong Road, Shanghai, 200093 China

## Abstract

Dual-band photodetection in mid- and near-wave infrared spectral bands is of scientific interest and technological importance. Most of the state-of-the-art mid-infrared photodetectors normally operate at low temperature and/or suffer from toxicity and high cost due to limitations of material properties and device structures. The capability of surface plasmons in confining electromagnetic waves into extremely small volume provides an opportunity for improving the performance for room temperature operation. Here, we report an *n-*InAsSb/*n-*GaSb heterostructure photodiode integrated with plasmonic two-dimensional subwavelength hole array (2DSHA) for room temperature two band photodetection. We demonstrate that with a properly designed 2DSHA, room temperature detectivities of the heterostructure device can be enhanced to ~1.4 × 10^9^ Jones and ~1.5 × 10^11^ Jones for the two bands peaked at 3.4 μm and 1.7 μm, respectively. In addition, we study the photocurrent enhancement in both photoconductor and heterojunction modes in the same integrated structure. The demonstration of single 2DSHA enhanced heterojunction photodiode brings a step closer to high sensitivity room temperature devices and systems which require multiband absorption.

## Introduction

Surface plasmon polaritons (SPPs)^1^ have gained much interest for their vast applications in extraordinary optical transmission^2,3^, cold atoms manipulations^4^, wavelength filtering^5^, plasmonic devices^6^, solar cell energy harvesting^7^, metamaterials^8^, ultrafast on-chip photonic information processing^9^, molecular sensing and spectroscopy^10,11^. Owing to plasmonic confinement that localizes light at the metal-dielectric interfaces, surface plasmons have been used for enhancing light absorption within the near-field region^12–16^. Plasmonic nanostructures such as two-dimensional subwavelength hole arrays (2DSHAs)^3,5,17^ have been integrated with infrared photodetectors based on InAs QDs^18–28^, InGaAs QW intersubband diode^29^ and quantum cascade structures^30^ for improving their device performances at cryogenic temperatures for single-band detection.

Multiple-band detection has been considered as the next generation infrared photodetectors^31–33^, as it allows for multiple information processing and improved target identification. Specifically, dual-band photodetection in mid-infrared (MIR, 3–5 µm) and near-infrared (NIR, 1–2 µm) spectral bands is crucial for communication, thermal imaging, medical treatment, remote sensing and military applications^34^. Current II-VI HgCdTe-based MIR/NIR dual-band photodetectors are reported to suffer from toxicity and high cost^34^. Recently, type-II InAs/GaSb/AlSb superlattice (T2SL) based dual- and multi-band photodetectors have been reported^35,36^, showing great prospects for sensitive focal planar arrays (FPAs) at low-temperature. Meanwhile, *n-*InAsSb/*n-*GaSb compact diodes have been investigated both in their physics and applications for MIR and NIR bands^37–41^. Owing to the broken bandgap alignment between InAsSb epitaxial layer and GaSb substrate, the properties of *n-*InAsSb/*n-*GaSb heterostructures can be tuned under different conditions^42,43^. Dual-band infrared photodetection based on *n-*InAsSb/*n-*GaSb heterostructure has been reported^44–46^ and their photoresponse characteristics can be tuned by bias voltage, doping, and temperature. Despite this flexibility, their sensitivities are still low due to small absorption space around the InAsSb-GaSb interface. An improvement in the detection efficiency is thus needed in order for them to operate at room temperature.

In this work, we design a single 2DSHA structure that can enhance light detections for both MIR and NIR bands in an *n-*InAsSb/*n-*GaSb heterostructure. There are three advantages for this material system. The first is the usefulness of the two absorption bands peaked at 3.4 μm and 1.7 μm which are in MIR and NIR, respectively. The second is the easy realization of lattice matching between the two materials. The third is the availability of the plasmonic 2DSHA with resonances in the two bands. We demonstrate that by selecting a 2DSHA whose SPR modes match with the absorption bands of InAsSb and GaSb, 2–3 times enhancement in performance can be achieved with detectivities of ~1.4 × 10^9^ Jones (for MIR band) and ~1.5 × 10^11^ Jones (for NIR band) at room temperature, which are comparable to the state-of-the-art for each band. The interaction between surface plasmons and photocarriers generation-recombination (g-r) is studied based on 2DSHA *n-*InAsSb/*n-*GaSb devices integrated in transverse and longitudinal configurations, from which some valuable principles in design of plasmon-enhanced photodetectors are proposed.

## Results

### Design of 2DSHA *n-*InAsSb/*n-*GaSb heterostructure

In the two-dimensional periodic metal nanohole structure, the interaction between light and the surface plasmons obeys momentum conservation given by ${k}_{spp}=|{k}_{x}+{G}_{i,j}|=|{k}_{0}\,\sin \,\theta +i{G}_{x}+j{G}_{y}|$, where *k*_0_sin*θ* is the in-plane incident wave vector and ${G}_{x}={G}_{y}=2\pi /p$ are the reciprocal lattice vectors. *k*_*spp*_ is the wave vector of the SPP at the gold-dielectric interface, which according to SPP dispersion relations^47–49^ can be expressed as ${k}_{spp}={k}_{0}\sqrt{{\varepsilon }_{m}{\varepsilon }_{d}/({\varepsilon }_{m}+{\varepsilon }_{d})}$. In the case of normal incidence *θ* = 0, the surface plasmon resonance (SPR) modes of 2DSHA are

1 $${\lambda }_{i,j}=p{\rm{Re}}[\sqrt{{\varepsilon }_{m}{\varepsilon }_{d}/({\varepsilon }_{m}+{\varepsilon }_{d})}]/(\sqrt{{i}^{2}+{j}^{2}}).$$

The (*i*, *j*) are set of integers denoting mode orders in the *x*- and *y*- directions, *ε*_m_ is the permittivity of gold that can be expressed by Drude model^50^ with the plasma frequency of *ω*_p_ = 1.37 × 10^16^ rad/s and collision frequency of *ω*_c_ = 4.07 × 10^13^ rad/s, and *ε*_*d*_ ~ 14.1 is the real permittivity of InAsSb (The imaginary part is ignored as it is much smaller than the real part). Note here that under oblique incidence, additional in-plane wavevector ${{\rm{k}}}_{0}{\rm{\sin }}{\rm{\theta }}$ will lead to shift for these SPR modes. For example, if this ${{\rm{k}}}_{0}{\rm{\sin }}{\rm{\theta }}$ has the samle direction with G_i,j_, the SPR modes would be red-shifting. This has been studied by the pioneering work related to extraordinary optical transmission in 2DSHAs^51^. Therefore, the SPR modes $({\lambda }_{{ij}})$ covering multiple absorption bands in a heterostructure photodiode can be designed with proper periodicity *p*. In the following, we illustrate the use of multiple resonances for achieving plasmon-enhanced multiband photodetection. The fundamental resonance mode is first matched with the InAsSb absorption band in MIR (~3.4 µm), where the periodicity of the 2DSHA is set to *p* = 900 nm to achieve *λ*_±1,0_ = *λ*_0,±1_ = 3.4 µm. The higher order resonances associated with the same periodicity (*λ*_±1,±1_ = 2.4 µm and *λ*_±2,0_ = *λ*_0,±2_ = 1.7 µm) are then employed to enhance absorptions in other photodetection bands located at shorter wavelength range. This can be achieved in a semiconductor with a bandgap larger than InAsSb and absorption band matching the higher order modes of the 2DSHA. For this, GaSb is an excellent candidate since its bandgap at 1.7 µm matches with the (±2, 0) mode of the 2DSHA. More importantly, InAsSb can be lattice-matched to GaSb when the composition of Sb is ~9% (InAs_0.91_Sb_0.09_). By further considering ohmic contact requirements in realistic device fabrication, we can therefore grow an unintentionally doped *n*-type InAs_0.91_Sb_0.09_ layer on an *n*-type GaSb substrate by molecular beam epitaxy (MBE) to form an *n*-InAsSb/*n-*GaSb heterostructure photodiode. As the bandgap of GaSb is larger than InAsSb, it would be ideal to employ backside illumination for our devices configuration. However, in order to directly demonstrate the contribution of plasmonic resonances in the metal nanostructure, front illumination was adopted instead. It should be noted that actually InAsSb layer can absorb 1.7 µm radiation, but owing to its very large absorption coefficient, the carrier surface recombination is very large, resulting in inefficient detection of 1.7 µm radiation. Therefore, we choose the lattice-matched GaSb as the absorption layer for the 1.7 µm band.

**Figure 1:**
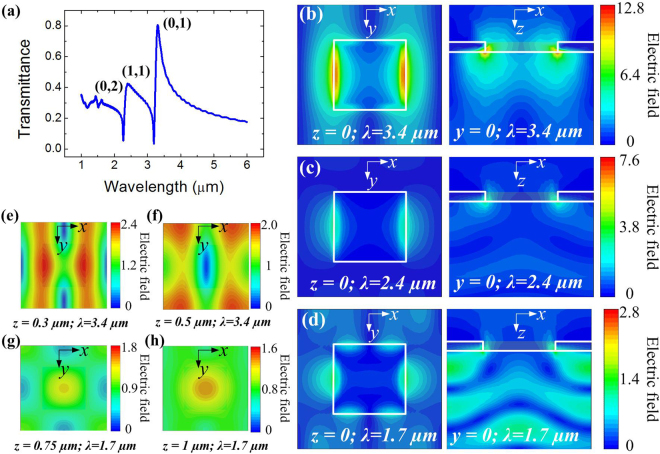
Simulation for the Au 2DSHA with *p* = 900 nm on the *n-*InAsSb/*n-*GaSb sample. (**a**) Simulated transmittance spectrum of the Au 2DSHA at 1–6 µm. (**b**–**d**) Electric field distributing patterns of *x-y* (*z* = 0) and *x-z* (*y* = 0) planes at the (**b**), fundamental, (**c**) second and (**d**), third modes. (**e**,**f**) Electric field distributing patterns of *x-y* plane for the fundamental mode at (**e**), *z* = 0.3 µm and (**f**), *z* = 0.5 µm. (**g**–**h**) Electric field distributing patterns of *x-y* plane for the third mode at (**g**), *z* = 0.75 µm and (**h**), *z* = 1 µm.

Owing to strong plasmonic light localization at the interface between metal and dielectric, thickness of the InAsSb film needs to be designed to ensure light absorption inside the InAsSb and GaSb. Figure 1 presents numerical analysis for the gold 2DSHA structure (as shown in Supplementary Fig. S1, the hole width *w* is optimized as half of the period *p*) (Supplementary information). The sharp peaks in the transmittance spectrum (Fig. 1a) denote the (0, 1), (1, 1), and (2, 0) SPR modes with their positions indicated by Equation 1. Figure 1(b–d) plots E-field distributions corresponding to (0, 1), (1, 1) and (2, 0) modes in *x-y* (*z* = 0) and *x-z* (*y* = 0) planes. At these modes, the electric fields in the semiconductor layers become large, leading to the increase of light absorption provided that they meet with the two bands. This will facilitate the generation of photoexcited electron and hole pairs (EHPs) resulting in photocurrent enhancement. Maximum electric field enhancement of 12.8, 7.6, and 2.8 can be achieved respectively for the fundamental, second, and third mode, which correspond to maximum relative power densities (proportional to E^2^) of 163.8, 57.8, and 7.8. Noted here that the fundamental and third modes overlap well with the absorption of MIR (InAsSb) and NIR (GaSb), respectively. Thus, it is possible to achieve plasmon-enhanced dual-band photodetection.

As mentioned, the dual-band photodetection originates mainly from absorption by InAsSb and GaSb around the interface. Therefore, we present E-field distributions for fundamental and third modes at different positions (Fig. 1(e,f)). As shown, for the mode at *λ* = 3.4 µm, it still has an electric field (relative power density) of 2.4 (6.0) and 2 (4) at *z* = 0.3 µm and *z* = 0.5 µm, respectively, indicating strong light-semiconductor interaction in the near field region. This electric field enhancement can also be observed in near field region from the third mode (*λ* = 1.7 µm). As evident from Fig. 1(g,h), the maximum electric field (relative power density) of 1.8 (3.2) and 1.6 (2.0) can be achieved at *z* = 0.75 µm, and *z* = 1 µm at *λ* = 1.7 µm, respectively. Therefore, by integrating this 2DSHA gold hole array on top of the heterostructure *n-*InAsSb/*n-*GaSb, the light intensity can be expected to become large in the two absorption layers, which will lead to increasing of photocurrents at the two bands. After considering the above near field analysis, we then designed thickness of InAsSb layer as 800 nm, and GaSb active absorption layer as 500 nm to make sure that the optical field are both enhanced in the dual-band semiconducting absorption layers.

**Figure 2:**
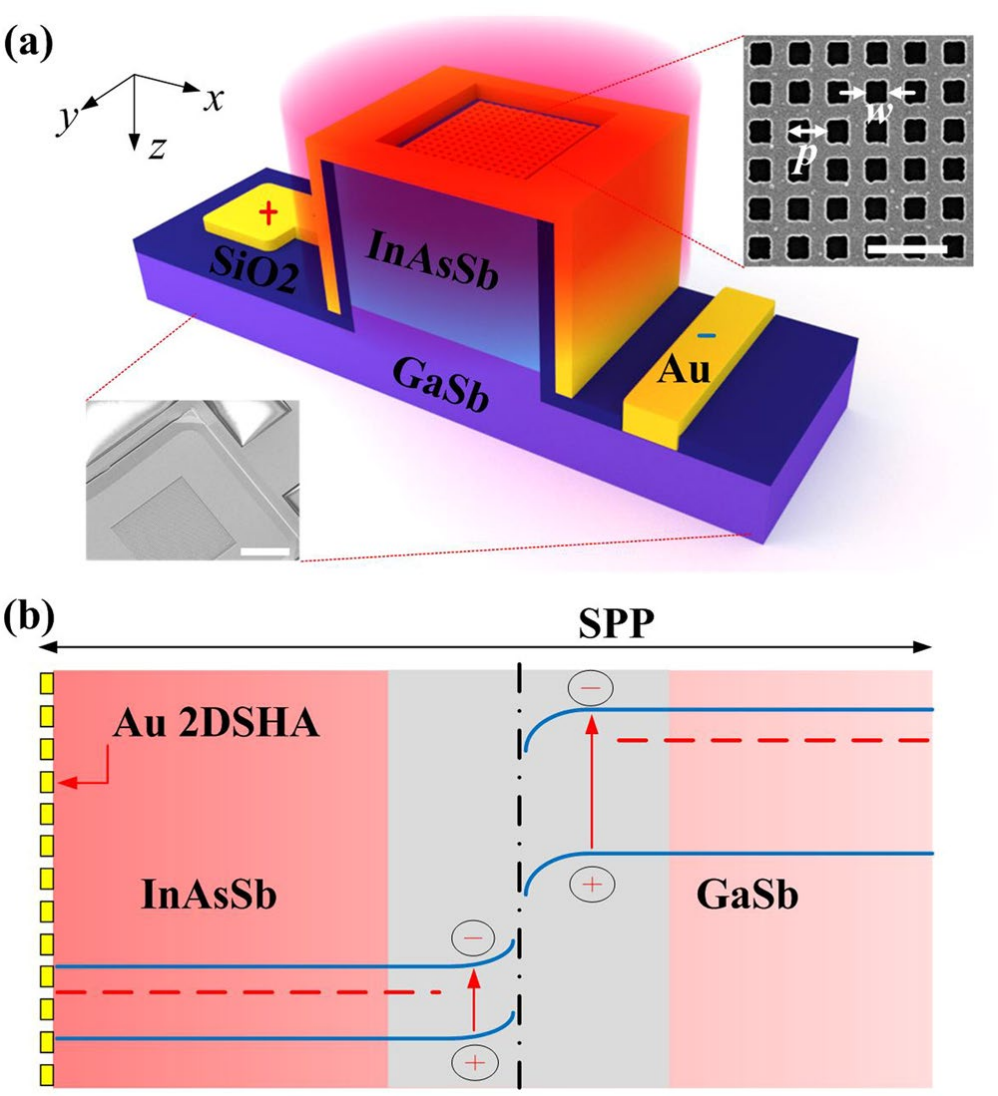
Plasmonic 2DSHA *n-*InAsSb/*n-*GaSb heterostructure for dual-band enhanced photodetection. (**a**) Schematic of the 2DSHA *n-*InAsSb/*n-*GaSb heterostructure photodiode (not to real scale). Thicknesses of the epitaxial InAsSb layer and the active absorption GaSb layer are 0.8 µm and 0.5 µm, respectively. The structure is designed for MIR and NIR dual-band photodetection from absorption of InAsSb and GaSb, respectively. The top inset is the scanning electron microscope (SEM) image of the 2DSHA with a period *p* and a hole width *w* (the scale bar represents 2 µm) while the bottom inset is SEM image (30° view of top of structure) of the 2DSHA heterostructure (the scale bar represents 100 µm). (**b**) Schematic of the energy band diagram of the 2DSHA *n-*InAsSb/*n-*GaSb heterostructure under forward bias for dual-band photodetection. The grey area represents the main absorption region by InAsSb and GaSb, the area with gradually varied red color represents the intensity of SPPs.

The lattice-matching between the epitaxial InAsSb layer and the GaSb substrate is confirmed by X-ray diffraction (XRD), while the absorption bands of InAsSb and GaSb were found to match well with the two SPR modes by normal photoluminescence and/or transmittance spectrum^46,52^. The schematic of the 2DSHA *n-*InAsSb/*n-*GaSb heterostructure is shown in Fig. 2a, with the top-view SEM image of the structure and the 2DSHA shown as insets. The detailed fabrication steps of the 2DSHA *n-*InAsSb/*n-*GaSb heterostructure are as shown in Supplementary Fig. S2. First, 300 × 300 µm^2^ mesa of 1.3 µm thickness (0.8 µm InAsSb and 0.5 µm GaSb) was defined by dry etching (Inductively Coupled Plasma-Reactive Ion Etching, ICP-RIE), followed by SiO_2_ deposition (Chemical Vapor Deposition, CVD) for passivation and Ti/Au (30 nm/300 nm) deposition (Electron Beam Evaporation) to form metal contacts after opening windows in SiO_2_ by normal RIE. The gold 2DSHA was then fabricated atop the InAsSb layer through electron beam lithography (EBL) and standard lift-off process. The 2DSHA consists of 60-nm thick gold with 5-nm thick titanium as the adhesion layer. The 60-nm gold thickness was chosen to exceed the skin depth (~4.3–10.7 nm for gold for *λ* = 1–6 µm) in order to avoid direct transmission through metal films^53^. This thickness also corresponds to the optimum thickness for having plasmonic oscillations at low damping loss, as illustrated in the mapping of 2DSHA transmittance in Supplementary Fig. S3. The hole width *w* is designed as half of the period *p*, in order to have the largest transmission at those resonance wavelengths (Supplementary Fig. S3)^17^. The 2DSHA also possesses polarization independence on the incident light (Supplementary Fig. S3), which offers convenience in real-applications^54^. In Supplementary Fig. S4, the optimization process for the periodicity *p* is presented. By fixing the periodicity to *p* = 900 nm, one can see that the SPR modes can match well with the two absorption bands at 3.4 µm and 1.7 µm. In addition to this, the 2DSHA allows the largest optical field enhancement in the photodiode, which translates to the best performance for the integrated heterostructure photodiode. Figure 2b shows schematic of the band energy diagram of the heterostructure under forward bias for dual band photodetection^46^. This simple diode has a type-III band gap line up, which makes the transportation of carriers very complicated. The dual-band detection originates from the absorption of photons in GaSb and InAsSb around the interface. The nanostructure integrated on surface of the diode can launch strong light confinement in semiconductor layers, which facilitates absorption of photons resulting in more photo-excited EHPs and thus enhancement in photocurrent.

### Characterization of 2DSHA *n-*InAsSb/*n-*GaSb heterostructure

**Figure 3:**
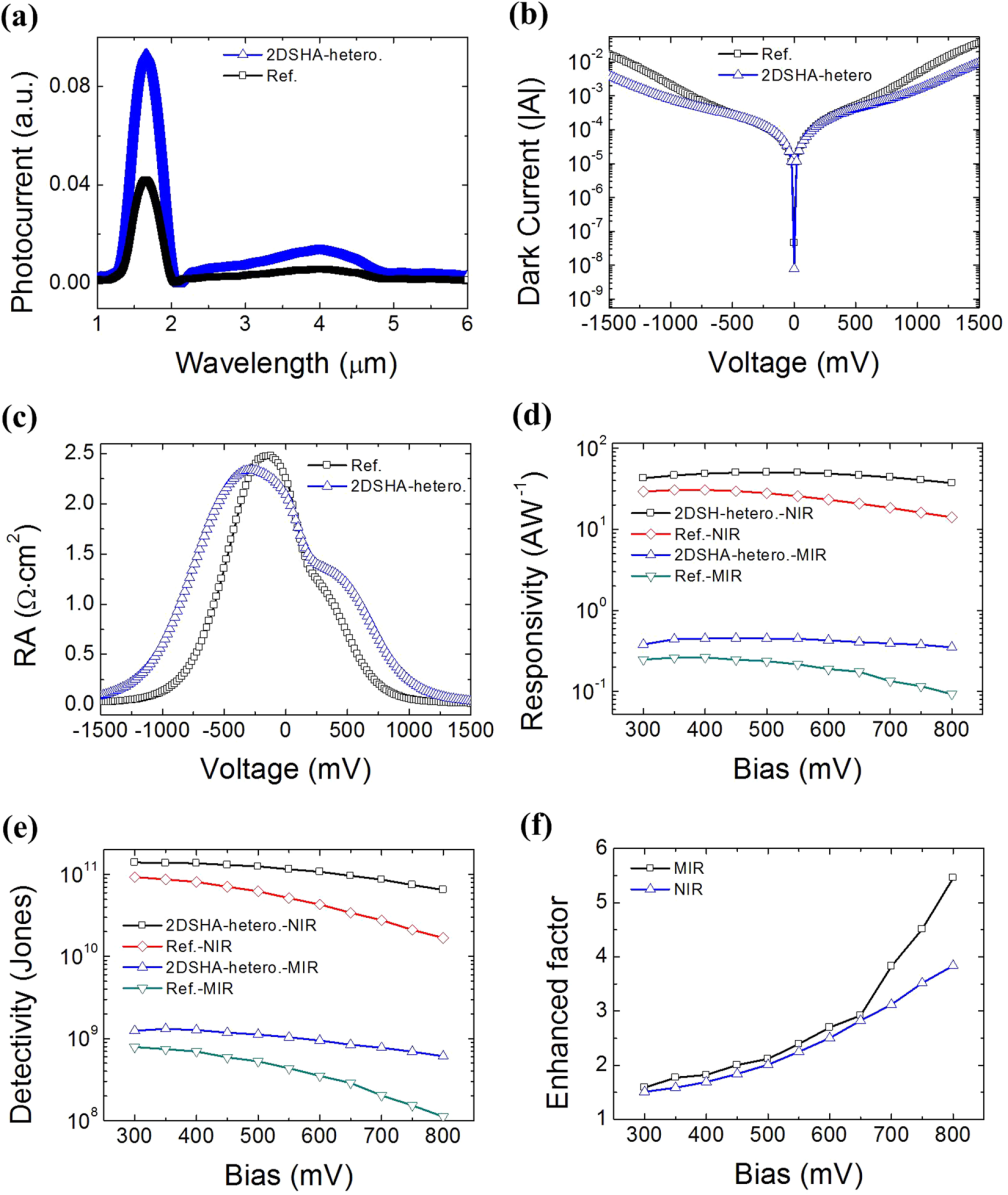
Characterization of the 2DSHA *n-*InAsSb/*n-*GaSb heterostructure and the reference without the 2DSHA. (**a**) Photocurrent spectra of the devices at 1–6 µm under 500 mV bias. (**b**) Dark current of the devices measured in the voltage range from −1500 mV to 1500 mV. (**c**) RA product of the devices in the voltage range from −1500 mV to 1500 mV. (**d**) Responsivities of the devices at MIR and NIR bands in the voltage range from 300 mV to 800 mV. (**e**) Detectivities of the devices at MIR and NIR bands from 300 mV to 800 mV. (**f)** Enhanced factor for the detectivity of the 2DSHA *n-*InAsSb/*n-*GaSb heterostructure compared to the reference at MIR and NIR bands.

Prior to evaluating dual band photocurrent spectra of the photodiode, we first characterized the transmittance spectrum of the gold 2DSHA on *n-*InAsSb/*n-*GaSb (Supplementary information). As evident from Supplementary Fig. S5, the 2DSHA on the *n-*InAsSb/*n-*GaSb with *p* = 900 nm exhibits fundamental (second/third) SPR wavelength at ~3.5 μm (~2.7 µm/1.8 µm), in good agreement with calculated values with Eq. 1 and shown in Fig. 1a. The slight red-shift in experiments is attributed to the presence of the scattering losses associated with hole roughness^55,56^ and the off-normal incidence angles from the condenser in the experimental set-up. Based on this 2DSHA design, we are able to make the resonance modes overlap with the two spectral bands. The spectral photocurrent characterization of the 2DSHA *n-*InAsSb/*n-*GaSb heterostructure was carried out in a standard commercial Fourier Transform Infrared Spectrometer (FTIR) setting (Supplementary information). In Fig. 3a, room temperature photocurrent spectrum taken from the 2DSHA *n-*InAsSb/*n-*GaSb heterostructure (blue curve) is presented over the wavelength range of 1–6 µm, while that from the bare *n-*InAsSb/*n-*GaSb heterostructure (black curve) is shown as reference. The heterostructure exhibits the ability of dual-band photodetection with peak photocurrent at ~3.4 µm (MIR) and ~1.7 µm (NIR), respectively. The 2DSHA *n-*InAsSb/*n-*GaSb heterostructure photodetector is found to have 2–3 folds of photocurrent enhancement at both the MIR and NIR range.

We then evaluate the performance of the devices at room temperature. The responsivity (*R*_i_) represents the performance of photoelectric conversion and is defined as:

2 $${R}_{i}={I}_{ph}/(PS)$$

with the unit of AW^−1^, where *I*_ph_ is the photocurrent, *P* is the incident light power density and *S* is the active absorption area. Generally, the noise equivalent power (NEP) and detectivity (D) are used to describe the minimum detectable power that a photodetector is able to response. The NEP is defined as the incident power when the output current equals the noise current, which can be expressed as NEP = *(SΔf)*^*1/2*^*/D*, where *Δf* is the electrical bandwidth. For our heterostructure photodiodes at room temperature, the noise from the dark current is the major contribution, therefore, *D* can be expressed as^34^:

3 $$D={R}_{i}/{(2qj+4kT/(RA))}^{1/2}$$

where *j* is the dark current density, *k* is the Boltzmann constant, *T* is the temperature, *R* is the dynamic resistance, and *A* is the area of the interface. Therefore, to evaluate final *D* of the heterostructure photodiodes, we first need to know the dark current characteristics.

Figure 3b shows dark current (*I-V)* of the 2DSHA *n-*InAsSb/*n-*GaSb heterostructure under voltage bias from −1.5 V to 1.5 V, where the dark current of the reference which is the same *n-*InAsSb/*n-*GaSb photodiode without the 2DSHA (black curve) is also presented for comparison. The dark current of the 2DSHA *n-*InAsSb/*n-*GaSb heterostructure is slightly smaller than that of the reference one, which may be attributed to reduced surface states. As shown in Fig. 3c, the RA product larger than 1 Ω·cm^2^ is generally obtained for bias voltages smaller than 500 mV, demonstrating low dark current characteristic for these two photodiodes.

A 1000 K blackbody radiation source was used to measure the photocurrents at room temperature under different voltage biases (Supplementary information). The responsivities of the photodiodes derived at MIR and NIR bands are shown in Fig. 3d. It is seen that the responsivities of both the 2DSHA *n-*InAsSb/*n-*GaSb heterostructure and the reference devices are two orders of magnitude higher in the NIR band than in the MIR band, and that the responsivities of the 2DSHA *n-*InAsSb/*n-*GaSb heterostructure device in MIR and NIR bands are always higher than those of the reference. Specifically, responsivities larger than 40 AW^−1^ and 0.3 AW^−1^ are generally achieved for the 2DSHA heterostructure photodetector at MIR and NIR bands, respectively, from 300 mV to 800 mV bias. This demonstrates the excellent photoelectric conversion nature of the integrated plasmonic device. Finally, the deduced detectivities of the devices versus bias voltage from 300 mV to 800 mV are shown in Fig. 3e. The detectivity of the two devices at both MIR and NIR bands slightly decreases when the bias voltage is increased. At 300 mV bias, the detectivity of the 2DSHA *n-*InAsSb/*n-*GaSb heterostructure photodiode is 1.4 × 10^9^ Jones at MIR and 1.5 × 10^11^ Jones at NIR, respectively. These values are comparable to the state-of-the-art single-band MIR and NIR room temperature photodetectors (Table S1), which are promising in the context of highly sensitive room temperature dual-band FPAs.

Since the *n-*InAsSb/*n-*GaSb heterostructure has a type III bandedge line-up, the accumulation and depletion of carriers around the interface can be significantly modified by varying the bias voltage. This suggests that the interaction between surface plasmons and absorption layers can be tuned, thus making the enhancement electrically tunable. This is illustrated in Fig. 3f, where the increase of bias from 300 mV to 800 mV corresponds to the increase in enhancement factor from ~1.5 times (1.5) to ~5.5 (3.8) times for the MIR (NIR) bands. This active tuning characteristic in 2DSHA heterostructure device provides additional advantages and flexibilities for potential applications.

### Interaction between EHPs and SPPs

To further understand the way SPP interacts with the active layer, we also fabricated InAsSb-based 2DSHA photoconductors using the same 2DSHA design (with *p* = 900 nm), but with the two metal contacts in transversal configuration (both metal contacts are on the InAsSb surface). The measurements were performed under both front-side and back-side illuminations (as illustrated in Supplementary Fig. S6). For the front-side illumination (Supplementary Fig. S7(a)), the photocurrent of 2DSHA photoconductor is much smaller than that of the bare photoconductor. For the back-side illumination (Supplementary Fig. S7(b)), however, we observed ~20% photocurrent enhancements in the 2DSHA photoconductor. The enhancement can be attributed to the reflection from the metallic 2DSHA on top of the InAsSb layer as the thickness of InAsSb is shorter than its absorption length (~1/(3000 cm^−1^) = 3.3 µm)^57^. These experimental findings suggest that the 2DSHA on an *n-*InAsSb photoconductor cannot give photocurrent enhancement despite the strong light confinement in 2DSHA.

**Figure 4:**
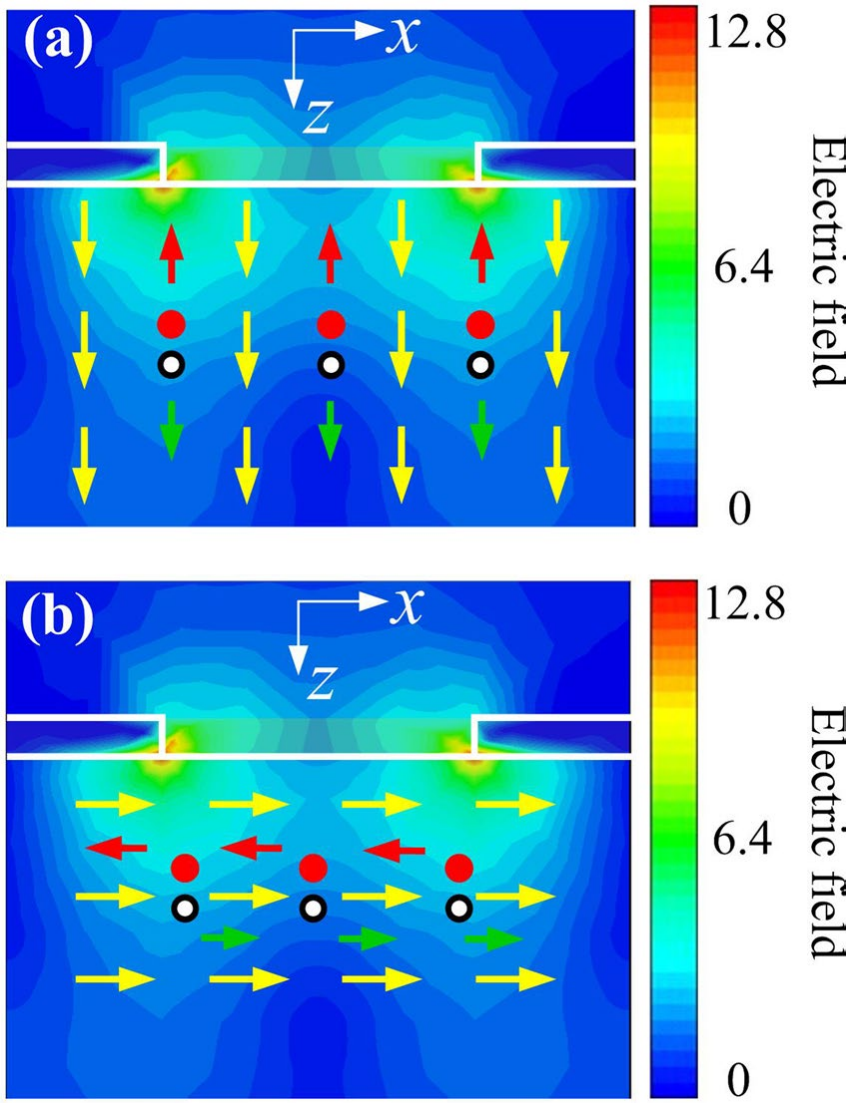
Interaction between EHPs and SPPs in (**a**) 2DSHA *n-*InAsSb/*n-*GaSb heterostructure and (**b**) 2DSHA *n-*InAsSb photoconductor. The yellow arrows represent the electric field by external bias. And the red and green arrows represent the drifting of the photoexcited electrons and holes, respectively. The color distribution represents the optical electric field (light intensity).

To gain deeper insights, we present the simulated light intensity distributions of the 2DSHA at *λ* = 3.4 µm. As the incident light is resonant in 2DSHA, light is then confined around the metal-dielectric interface, generating strong optical electric fields which later translate to the increased photoexcited EHPs. The movement of these EHPs is governed by two mechanisms, namely diffusion caused by the gradient of carrier concentration; and drift caused by an external voltage bias. The ratio between lifetime and drift time (transit time) of these EHPs determines the final Gain of the detection and eventually the photocurrent. In case of the 2DSHA *n-*InAsSb/*n-*GaSb heterostructure (Fig. 4a), the external electric field (denoted by the yellow arrows) is in the same longitudinal direction (*z*-direction) as the diffusion movement. Thus, EHPs can be easily collected by electrodes because of very small recombination within the thin InAsSb layer (i.e., <0.8 µm). In case of the 2DSHA *n-*InAsSb photoconductor (Fig. 4b), the external electric field is along the transverse direction while the diffusion is in longitudinal direction. This means a lot of carriers will be recombined before being collected by electrodes due to the long transit time, which still holds true even after generation of EHPs is improved as 2DSHA is integrated onto the photoconductor. This seems to explain why there is no photocurrent enhancement in 2DSHA *n-*InAsSb photoconductor.

Indeed, the role of EHPs recombination is crucial for photocurrent enhancement in the above two configurations. In order to have 2DSHA device with the largest enhancement, care must be taken to ensure the following requirements. First, drift and diffusion should be in the same direction to allow fast collection by electrodes. Second, the carrier recombination needs to be minimized to ensure a full collection of photo-generated carriers. For transverse configuration, this means the transit time should be smaller than the carrier recombination lifetime. This can be achieved by either shrinking lateral size of the device or introducing additional conducting channels^58^. These requirements are satisfied in 2DSHA *n-*InAsSb/*n-*GaSb heterostructure configuration. In addition, the fact that electrodes are defined along longitudinal direction makes it possible to fabricate large FPAs. We believe these findings are of relevance for the design of plasmon-enhanced photodetectors and/or FPAs.

## Discussion

In conclusion, we have proposed and demonstrated a plasmonic enhancement for dual-band photodetection in an *n-*InAsSb/*n-*GaSb heterostructure photodiode by matching the SPR modes of a gold 2DSHA structure with the two absorption bands of *n-*InAsSb and *n-*GaSb. The highest achievable detectivities of 1.4 × 10^9^ Jones (for MIR band) and 1.5 × 10^11^ Jones (for NIR band) at 300 mV bias are comparable with those of the state-of-the-art single MIR and NIR room temperature photodetectors. We also have demonstrated the active tuning of the enhancement by changing the external voltage bias. By integrating 2DSHA in two electrode configurations, some valuable insights for designing plasmon-enhanced photodetector have been proposed. The novel integrated heterostructures have a lot of advantages such as easy material growth, room temperature operation, and the ability to extend to large arrays. Moreover, the first demonstration of the metallic 2DSHA enhanced dual-band heterostructure photodiode by different plasmonic resonance modes opens up a promising avenue of developing high sensitivity multiband room temperature FPAs.

## Methods

### Numerical simulations

The transmittance and E-field distributions of the gold 2DSHA were calculated by commercial software (CST Suite). The transmittance mapping for different gold thicknesses, angles, and hole widths are calculated by FDTD (FDTD solutions, Lumerical Inc). These two simulation tools got very consistent and confirmed results. For all the simulations in this work, Drude model was used for the permittivity of gold, the permittivity of InAsSb was from pulished data, and the boundary conditions were set to periodic boundary condition for the 2DSHA.

### Transmittance characterizations

The transmittance of the 2DSHA *n-*InAsSb/*n-*GaSb sample was obtained in the microscopy settings using infrared light source from the FTIR. The 2DSHA structure was fabricated on InAsSb surface, which is in 100 µm × 100 µm footprint. The transmitted light from 2DSHA structure is collected by 36x objective lens over 40 µm × 40 µm footprint into a built-in MCT detector (liquid N2 cooled) in the microscope. The transmission response is normalized to the transmission response of a bare *n-*InAsSb/*n-*GaSb sample.

### Photocurrent spectra measurements

The photocurrent spectra were measured in the FTIR settings, where the FTIR internal detector is replaced by our photodetector. The infrared light source is first processed inside the FTIR before it is focused onto the photodetector sample mounted on a XYZ stage outside FTIR. The electrical signal is then amplified by a low noise current preamplifier before being connected back to FTIR via its external port. In the experiments, the scanning frequency was set as 10 KHz owing to the fast response of this photon type heterostructure photodiode.

### Blackbody measurements

The blackbody response was obtained by directing a 1000 K blackbody radiation onto the 2DSHA *n-*InAsSb/*n-*GaSb and reference heterostructures through a chopper and a mid-IR or near-IR optical bandpass filter. The measured photocurrent was converted into photovoltage by a low-noise current preamplifier (Model SR570, Stanford Research Systems) and then readout by a SR810 Lock-in amplifier. A standard power meter (OPHIR PHOTONICs) was used to calibrate the irradiation power density (Fig. S8) of the blackbody radiation in free space.

### Data availability

All data generated or analyzed during this study are included in this published article (and its Supplementary Information files).

## Electronic supplementary material

Supplementary Information
