# Supplementary material for: Single Plasmonic Structure Enhanced Dual-band Room Temperature Infrared Photodetection
Source: Sci Rep. 2018 Jan 24;8:1548. doi: 10.1038/s41598-018-20028-6 (PMC5784088; doi:10.1038/s41598-018-20028-6)
Supplement: Supplementary file 1 — Supplementary Information [file 41598_2018_20028_MOESM1_ESM.pdf]

Single Plasmonic Structure Enhanced Dual-band Room Temperature Infrared Photodetection

Jinchao Tong[1], Landobasa Y. M. Tobing,[1], Yu Luo[1], Dawei Zhang[2] & Dao Hua Zhang[1*]

[1]School of Electrical and Electronic Engineering, Nanyang Technological University, 50 Nanyang Avenue, 639798, Singapore.

[2]Ministry of Education and Shanghai Key Lab of Modern Optical System, University of Shanghai for Science and Technology, 516 Jungong Road, Shanghai 200093, China.

Correspondence and requests for materials should be addressed to D.H.Z. (email: EDHZHANG@ntu.edu.sg)

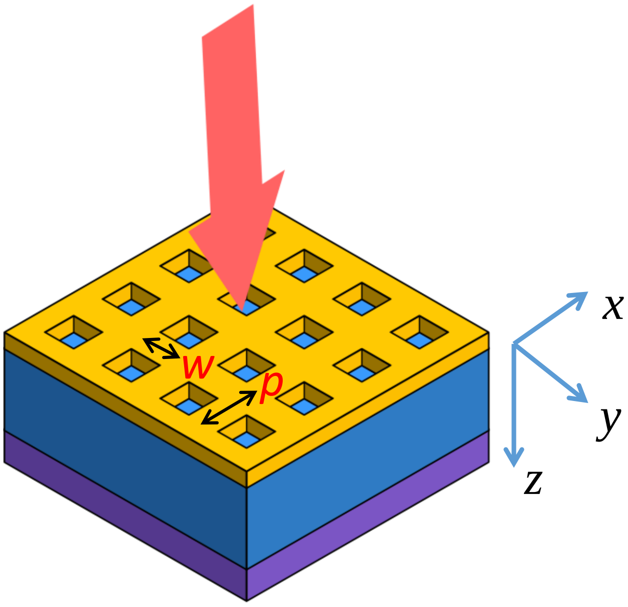

Figure S1. Schematic of the 2DSHA. *p* is the hole period and *w* is the hole width.

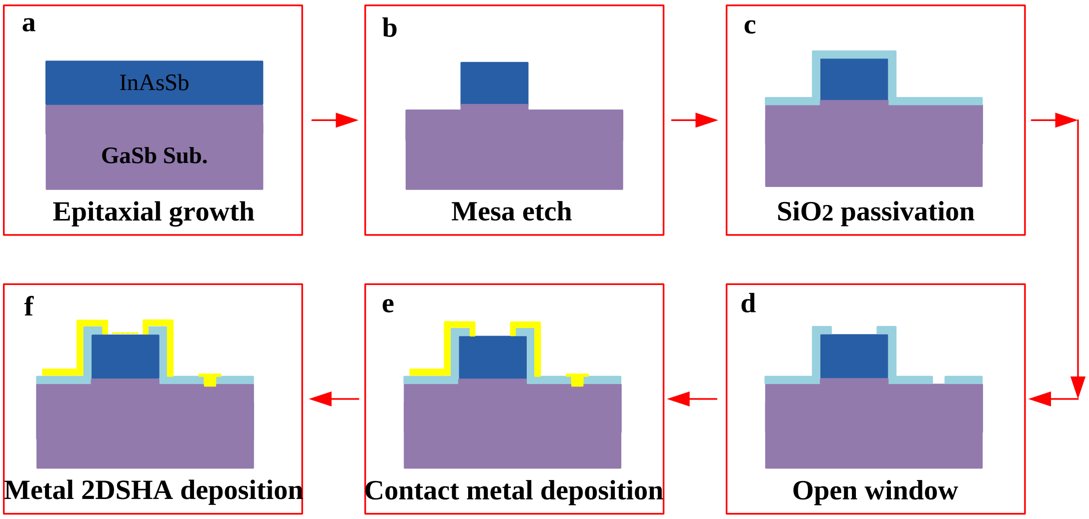

Figure S2. Schematic of fabricating procedure of the 2DHSA *n*-InAsSb/*n*-GaSb heterostructure. (a) Epitaxial growth. (b) Mesa etch. (c) SiO_2 passivation. (d) Open window. (e) Contact metal deposition. (f) Gold 2DSHA deposition.

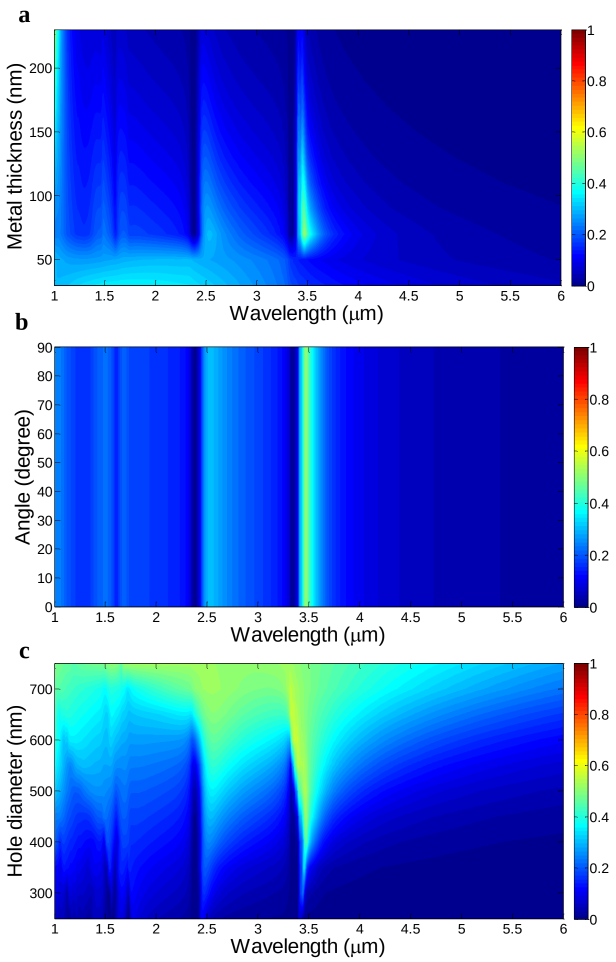

Figure S3. Design for the Au 2DSHA. Transmittance with respect to (a) gold thickness, (b) polarization angle and (c) hole width (diameter).

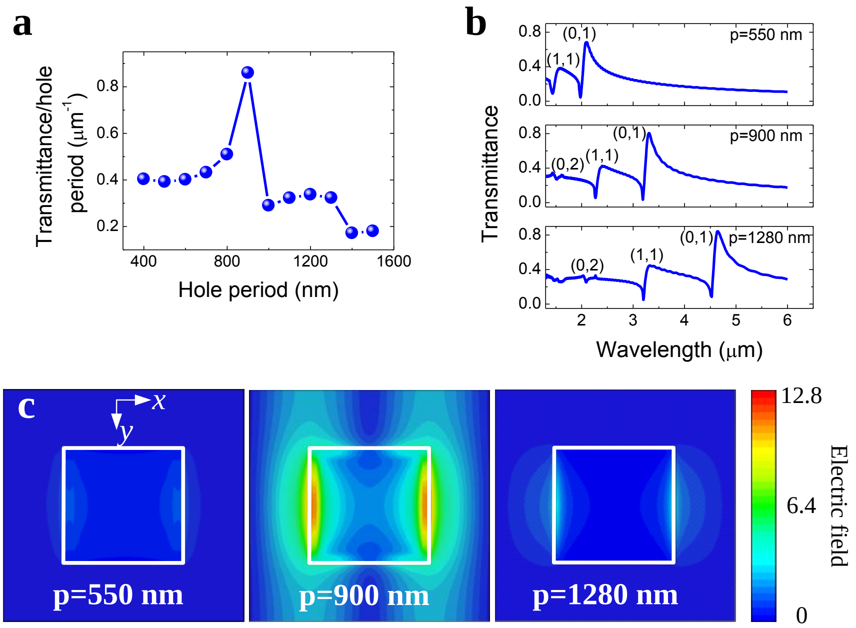

Figure S4. Optimization for the periodicity of the gold 2DSHA. (a) Transmittance/hole period ratio with respect to the hole period in 2DSHA. The width of the hole is half of the hole period. (b) Simulated transmittance of 2DSHAs with different hole period. (c) Electrical field distributions in x-y plane (z=0) for 2DSHAs with different hole period.

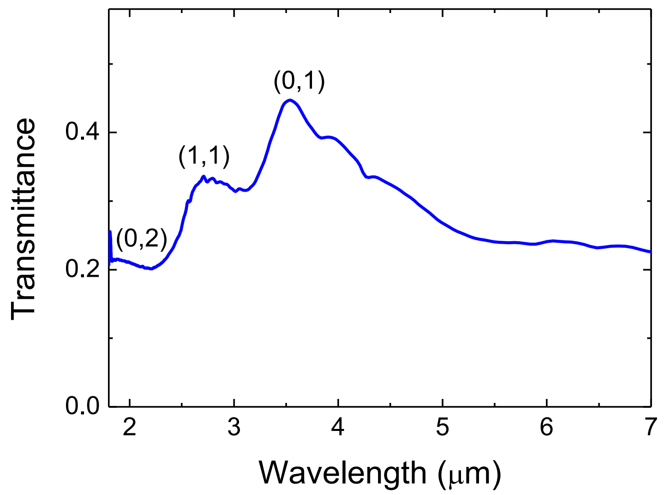

Figure S5. Measured transmittance spectrum of the Au 2DSHA on *n*-InAsSb/*n*-GaSb.

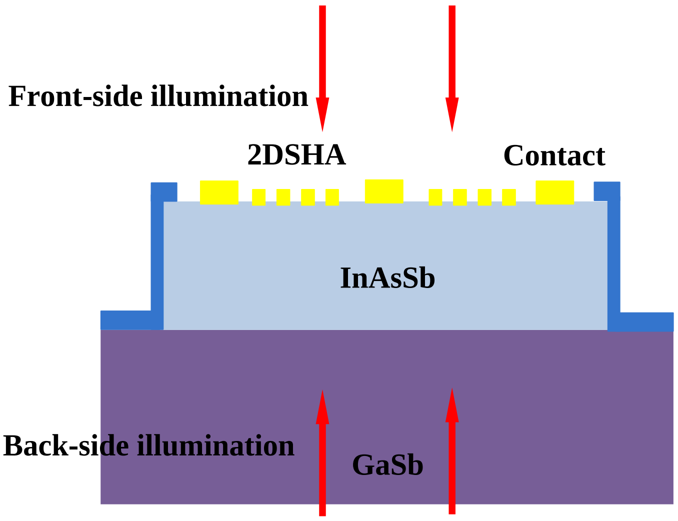

Figure S6. Schematic of the 2DSHA *n*-InAsSb photoconductor.

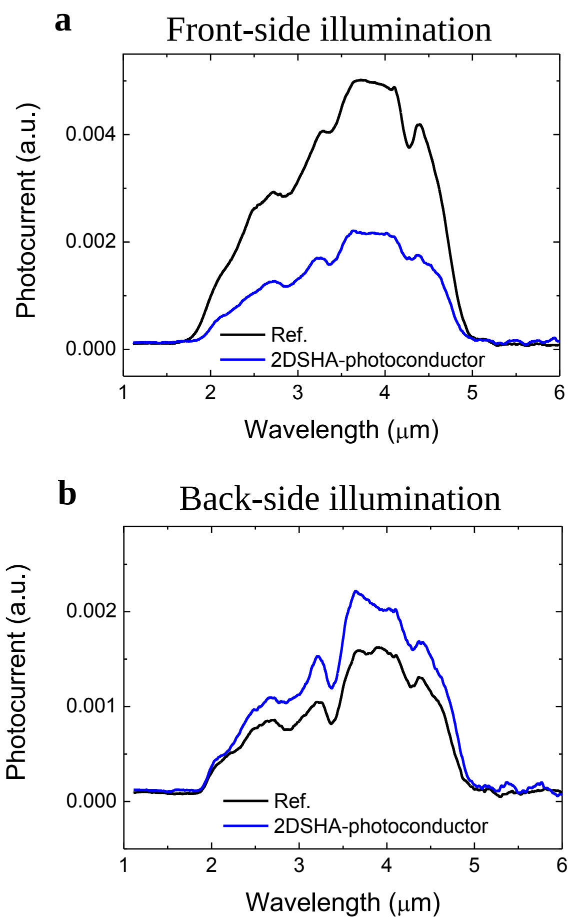

Figure S7. Photocurrent spectra of the 2DSHA *n*-InAsSb photoconductor and the reference under (a) front-side illumination and (b) back-side illumination.

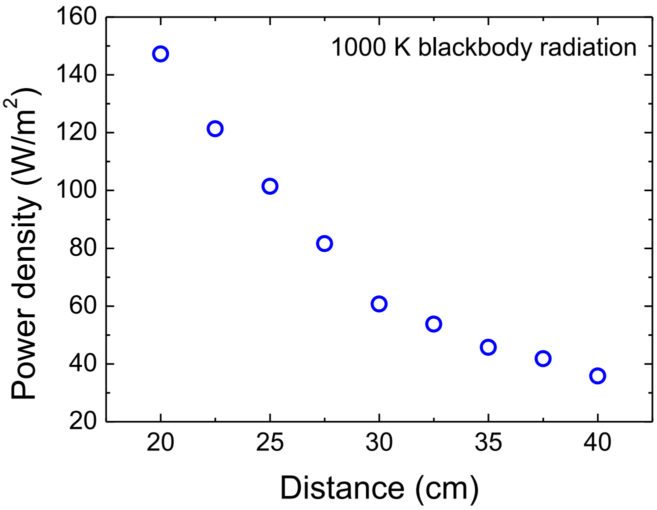

Figure S8. Calibrated power density of the 1000 K blackbody radiation source in free space.

Table S1 A summary of room temperature one-band MIR and NIR photodetectors. The performance at each band of our devices is comparable to that of reported single band photodetectors.

Photodetector (MIR)	Detectivity (10^9Jones)	Bias (|V|)	Ref.	Photodetector (NIR)	Detectivity (10^{11}Jones)	Bias (|V|)	Ref.
2DSHA *n*-InAsSb/*n*-GaSb	1.4	0.3	This work	2DSHA *n*-InAsSb/*n*-GaSb	1.5	0.3	This work
InAsSb PV	0.8-1	1	2	InGaAs PIN	3-10	0.5	1
InAs PV	4.5	0.5	3	GaSe/GaSb	4	-	8
PbSe PC	2.5	15	4	Perovskite/Conjugated-Polymer Composite	0.032	1	9
MCT PC	4	-	5	Graphene/Crystalline Silicon Schottky	3.9	0	10
InAsSb nBn	1	1	6	*n*-type β-FeSi2/pβ-FeSi2/p-type Si heterojunction	0.015	1	11
BP-As	4.9	-	7	GaAs Nanocone Array/Monolayer Graphene Schottky	1.83 (850 nm)	0	12

Supplementary Reference

1. http://www.hamamatsu.com/resources/pdf/ssd/g12181_series_kird1117e.pdf, accessed on 01/11/2017.
2. http://www.hamamatsu.com/resources/pdf/ssd/p13243_series_kird1130e.pdf, accessed on 01/11/2017.
3. http://www.hamamatsu.com/resources/pdf/ssd/p10090-01_etc_kird1099e.pdf, accessed on 01/11/2017.
4.https://www.thorlabs.com/newgrouppage9.cfm?objectgroup_id=6479, accessedon 01/11/2017.
5. http://irassociates.com/, accessedon 01/11/2017.
6. Alexander Soibel, Cory J. Hill, Sam A. Keo, Linda Hoglund, Robert Rosenberg, Robert Kowalczyk, Arezou Khoshakhlagh, Anita Fisher, David Z.-Y. Ting, and S. D. G. Room temperature performance of mid-wavelength infrared InAsSb nBn detectors. *Appl. Phys. Lett.* **105,** 23512 (2014).
7. Long, M. *et al.* Room temperature high-detectivity mid-infrared photodetectors based on black arsenic phosphorus. *Sci. Adv.* **3,** e1700589 (2017).
8. Wang, P. *et al.* Arrayed Van Der Waals Broadband Detectors for Dual-Band Detection. *Adv. Mater.* **29,** 1604439 (2017).
9. Chen, S. *et al.* A Flexible UV–Vis–NIR Photodetector based on a1. Chen, S. et al. A Flexible UV–Vis–NIR Photodetector based on a Perovskite/Conjugated-Polymer Composite. Adv. Mater. 28, 5969–5974 (2016). Perovskite/Conjugated-Polymer Composite. *Adv. Mater.* **28,** 5969–5974 (2016).
10. Lv, P., Zhang, X., Zhang, X., Deng, W. & Jie, J. High-sensitivity and fast-response graphene/crystalline silicon schottky junction-based near-IR photodetectors. *IEEE Electron Device Lett.* **34,** 1337–1339 (2013).
11. Shaban, M., Nomoto, K., Izumi, S. & Yoshitake, T. Characterization of near-infrared n-type β-$FeSi_2$/p-type Si heterojunction photodiodes at room temperature. *Appl. Phys. Lett.* **94,** 222113 (2009).
12. Luo, L. B. *et al.* Near-infrared light photovoltaic detector based on GaAs nanocone array/monolayer graphene schottky junction. *Adv. Funct. Mater.* **24,** 2794–2800 (2014).
